# Correlations of strength, proprioception, and tactile sensation to return-to-sports readiness among patients with anterior cruciate ligament reconstruction

**DOI:** 10.3389/fphys.2022.1046141

**Published:** 2022-12-07

**Authors:** Xiaoli Ma, Lintao Lu, Zhipeng Zhou, Wei Sun, Yan Chen, Guofeng Dai, Cheng Wang, Lijie Ding, Daniel Tik-Pui Fong, Qipeng Song

**Affiliations:** ^1^ College of Sports and Health, Shandong Sport University, Jinan, China; ^2^ Department of Orthopedic Surgery, Qilu Hospital, Cheeloo College of Medicine, Shandong University, Jinan, China; ^3^ School of Sport, Exercise and Health Sciences, Loughborough University, Loughborough, United Kingdom

**Keywords:** anterior cruciate ligament reconstruction, neuromuscular control, strength, cutaneous sensation, ACL injury

## Abstract

**Objectives:** Anterior cruciate ligament reconstruction (ACLR) is the most common surgery for anterior cruciate ligament (ACL) injuries, and the relationships between patients’ return to sports (RTS) readiness and different physical functions are inconclusive among patients with ACLR. This study aimed to investigate the correlations of strength, proprioception, and tactile sensation to the RTS readiness among patients with ACLR.

**Methods:** Forty-two participants who received ACLR for at least 6 months were enrolled in this study. Their strength, proprioception, and tactile sensation were tested, and their RTS readiness was measured with the Knee Santy Athletic Return to Sports (K-STARTS) test, which consists of a psychological scale [Anterior Cruciate Ligament Return to Sports after Injury scale (ACL-RSI)] and seven functional tests. Partial correlations were used to determine their correlations while controlling for covariates (age, height, weight, and postoperative duration), and factor analysis and multivariable linear regressions were used to determine the degrees of correlation.

**Results:** Knee extension strength was moderately correlated with K-STARTS total, ACL-RSI, and functional scores. Knee flexion strength, knee flexion and extension proprioception, and tactile sensation at the fifth metatarsal were moderately correlated with K-STARTS total and functional scores. Strength has higher levels of correlation with functional scores than proprioception.

**Conclusion:** Rehabilitation to promote muscle strength, proprioception and tactile sensation should be performed among patients with ACLR, muscle strength has the highest priority, followed by proprioception, with tactile sensation making the least contribution.

## 1 Introduction

The anterior cruciate ligament (ACL) is one of the most important ligaments of the knee joint that prevents the anterior glide of the tibia, maintains the knee joint’s stability, and enables the human body to complete a variety of complex and challenging movements (Noyes et al., 2015). ACL injury is one of the most common and devastating knee injuries in rotational and contact sports (Paterno et al., 2010). More than 200,000 ACL tears was reported in the United States annually, particularly among athletes or recreational sportsmen (Lentz et al., 2012). ACL reconstruction (ACLR) is the most common surgery for ACL injuries, and it is considered effective in restoring the function of the knee joint (Leys et al., 2012). A previous study has shown that return to sports (RTS) rates are relatively low after ACLR. Although approximately 81% of individuals who underwent ACLR returned to some form of sports activities, only 55% returned to their pre-injury level of competition (Ardern et al., 2014b).

The Knee Santy Athletic Return to Sports (K-STARTS) test is considered an appropriate and objective measure of functional improvement after ACLR (Blakeney et al., 2018). The K-STARTS test consists of a psychological scale [ACL Return to Sports after Injury scale (ACL-RSI)] and seven functional tests. The ACL-RSI is specific to assess the psychological readiness for RTS after ACLR. Functional tests provide a comprehensive assessment of knee function and reflect the overall motor skill and performance of the lower extremities.

A variety of factors may influence RTS readiness, with inadequate neuromuscular control being at the forefront (Paterno et al., 2010). Decreased strength and impaired sensation are common in weeks, months, and even years after ACLR and may considerably affect the neuromuscular control during locomotion (Kennedy et al., 1982). The ability of muscles to generate adequate force is critical for neuromuscular control (Pijnappels et al., 2008). When an individual moves strenuously (e.g., change of direction, jump, etc.), the muscles around the knee joint work together to maintain the stability of the knee joint, improve neuromuscular control, and help individuals to safely return to the competition. Proprioception refers to the perception of one’s own body and movements through information generated inside the body, while tactile information, sensed by receptors in the skin, is concerned with sensory stimuli originating outside the body, such as the physical characteristics of the environment (Song et al., 2021). As primary components of the somatosensory system, proprioception and cutaneous sensation account for about 60–70% of balance control (Li et al., 2019).

The correlations of strength, proprioception, and tactile sensation with RTS readiness remain unclear, although they are considered the primary contributors to neuromuscular control (Song et al., 2021). Several previous studies have indicated that strength is correlated with functional tests (Xergia et al., 2015), and insufficient strength results in a high risk of re-injury (Thomee et al., 2011). Whether the strength is related to the psychological readiness measured by ACL-RSI and the overall RTS readiness measured by K-STARTS is unknown among patients with ACLR. Some studies showed that proprioception is correlated with functional tests (Katayama et al., 2004), whereas others showed non-significant correlations (Muaidi et al., 2009). The correlations of proprioception to psychological readiness for RTS still need to be further investigated. Literature on the correlations of tactile sensation to RTS variables is scarce. Strength training and sensory recovery have been applied to the rehabilitation among patients with ACL injury (Kruse et al., 2012), the necessity of these rehabilitation programs cannot be determined without examining the correlations of strength and sensation with RTS variables. In addition, one study showed a strong correlation between muscle strength and functional performance (*r* = 0.649, *p* < 0.05) (Greenberger and Paterno, 1995), and another study detected a moderate correlation between proprioception and functional performance (*r* = −0.389, *p* < 0.05) (Katayama et al., 2004). No correlation was detected between cutaneous sensitivity and dynamic balance control (Song et al., 2021). To our knowledge, no one has yet to correlate strength, proprioception and tactile sensation with RTS among patients with ACLR in a single study, the priority of different rehabilitation approaches is difficult to be determined.

This study aimed to investigate the correlations of strength, proprioception, and tactile sensation with RTS variables in patients with ACLR. The following hypotheses were proposed: 1) Strength, proprioception, and tactile sensation are significantly correlated with K-STARTS, ACL-RSI, and functional tests, and 2) Higher levels of correlation existed between strength and RTS for proprioception, while the lowest levels existed between tactile sensations and RTS.

## 2 Materials and methods

### 2.1 Participants

An *a priori* power analysis (G*Power Version 3.1) indicated that a minimum of 33 participants are needed to obtain the alpha level of 0.05 and the statistical power of 0.80 based on a previous report, which detected a *r* ^2^ = 0.24 between strength and RTS among 78 young (13–30 years) patients who received ACLR (Della Villa et al., 2021). Patients with high sports demand who had underwent ACLR were enrolled in this study. The inclusion criteria were as follows: 1) aged 18–40 years; 2) regular participation in certain sports before the injury and willing to RTS after ACLR; 3) Tegner≥5; 4) unilateral ACL rupture and ACLR through arthroscopy; 5) without combined meniscus injury; 6) no other lesions; 7) 6–18 months after ACLR. The exclusion criteria were as follows: 1) associated knee ligamentous injuries within 3 months, 2) previous knee surgery, 3) clinically relevant cardiovascular history, 4) clinically relevant neurological and neuromuscular disorders and 5) associated organ diseases that cannot be tolerated. A total of 42 participants were enrolled after the eligibility assessment (female = 12, male = 30, age: 27.6 ± 6.8 years, height: 181.8 ± 9.0 cm, weight: 80.4 ± 8.9 kg, BMI: 24.4 ± 2.7, postoperative duration: 10.3 ± 3.8 months) and were included in the final analysis. Of them, 15, 11, 10, 5 and 1 participated in basketball, soccer, badminton, table tennis and fencing. Human participation was approved by Institutional Review Boards in Shandong Sport University (2022013) and was in accordance with the Declaration of Helsinki.

### 2.2 Protocol

The participants provided written informed consent and completed a battery of questionnaires, including the International Knee Documentation Committee questionnaire, Tegner, and Visual Analog Scale (VAS). The results were used to determine whether the participants should be included in the study. Multiple measures of strength, proprioception and tactile sensation tests were performed. The order of the K-STARTS, proprioception and tactile sensation tests is randomized and the muscle strength is tested at last to avoid fatigue.

### 2.3 K-STARTS test

**FIGURE 1:**
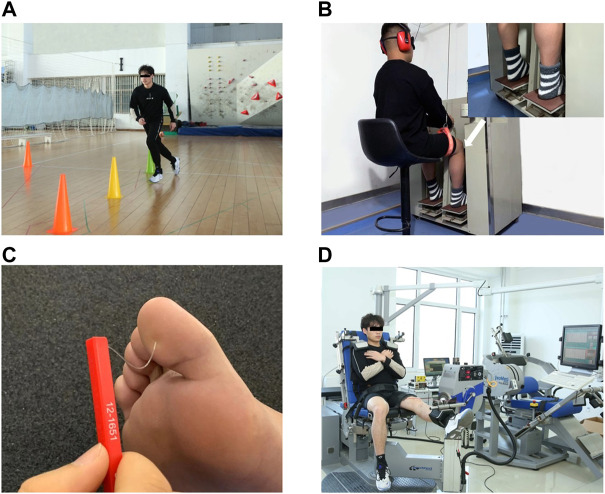
Test illustrations. **(A)** K-STARTS tests. **(B)** Proprioception test using a proprioception test device. **(C)** Tactile sensation test with a set of Semmes–Weinstein monofilaments. **(D)** Strength test using the IsoMed 2000 strength-testing system.

The K-STARTS test had high reliability (intraclass correlation coefficient (ICC) = 0.87, coefficient of variation = 7.8%) in assessing RTS readiness among patients with ACLR (Franck et al., 2021). It consists of an ACL-RSI scale and seven functional tests (Figure 1A). All tests were supervised by two trained research assistants. The ACL-RSI scale consists of 12 psychological questions. The participants were asked to rate the degree of fear for each task using VAS from 0 (no fear at all) to 10 (most fearful). Three points were awarded for ACL-RSI scores of 76% or higher, 2 points for scores between 64% and 75%, 1 point for scores between 56% and 63%, and 0 points for less than 55% (Franck et al., 2021).

The functional scores were determined as follows. 1) Single-leg landing: Participants stood on their affected leg for about 10 s, scored by the Qualitative Assessment of Single-leg Landing (QASLS). The QASLS score ranges between 0 (best) and 10 (worst). Participants with QASLS scores of 0, 1, 2, and ≥3 were given 3, 2, 1, and 0 points, respectively (Franck et al., 2021). 2) Dynamic valgus: a penalty of 3 points was applied if a participant was judged to have a dynamic valgus of the limb during the single-leg loading task. 3) Single, triple, side, and crossover hop tests: the jump distance of each leg in each of the four tests was measured. Hop tests were evaluated using the Limb Symmetry Index (the percentage deficit of the distance hopped on the involved leg compared with the uninvolved contralateral leg). Participants with ≥90%, 80%–90%, and ≤80% distance deficits were given 3, 2, and 1 point, respectively, and 0 point was attributed when pain prevented the test (Blakeney et al., 2018). 4) The Modified Illinois Change of Direction Test (MICODT): the participant sprinted forward as fast as he/she could, following the direction of the arrows and running around the markers. An average time of ≤12.5 s scored 3 points, 12.5–13.5 s scored 2 points, >13.5 s scored 1 point, and 0 point was attributed when pain prevented the test.

### 2.4 Proprioception test

The participant’s knee proprioception threshold was assessed using a proprioception test device (Sunny, AP-II, China; Figure 1B), which showed good test-retest reliability (ICC = 0.74–0.94) (Sun et al., 2015). The device consists of a platform and a pedal. Two electric motors drive the platform at an angular velocity of 0.4°/s. An electronic goniometer in the device recorded the angular displacement of the platform. All participants were seated on a height-adjustable chair with feet on the testing pedal during the test. The hip and knee joints were flexed 90°, respectively, the ankle joint was in a neutral position, and the lower leg was perpendicular to the surface of the platform. The participants wore eye masks and headphones and played soothing music in the headphones to reduce the visual and auditory interference caused by the external environment. Each test movement began by placing the foot on the horizontal platform (position of 0°). The experimenter presses the direction button, and the testing pedal will move in the corresponding direction. Participants were instructed to concentrate on their foot, press the hand switch to stop the movement of the platform when they could sense motion and say (point out) the direction of the joint motion. At least five trials were performed for each direction. The minimal three angles sensed in each direction were used for data analysis.

### 2.5 Tactile sensation test

The tactile sensation was assessed with a set of Semmes–Weinstein monofilaments (North Coast Medical, Inc., Morgan Hill, CA, United States; Figure 1C), which showed good test-retest reliability (ICC = 0.83–0.86) (Collins et al., 2010). Six monofilaments with different sizes were used in this study: 2.83 (0.07 g), 3.61 (0.4 g), 4.31 (2 g), 4.56 (4 g), 5.07 (10 g), and 6.65 (300 g). The filament size was log10 (10 × force in milligrams). During the assessment, all participants were supine with their eyes closed in a quiet environment. The filaments were randomly applied vertically to the skin on the bases of the great toe, first and fifth metatarsals, arch, and heel on the affected side until they were bent 90° for 1.0–1.5 s. Tactile sensitivity was determined by the initial application of the thin filaments, progressing to the thicker filaments until the participants would be able to detect the touch (Machado et al., 2017). Participants were asked to name the exact location where a monofilament was detected. If a monofilament was perceived correctly on a test location, the tactile sensation threshold was recorded. If a monofilament was not perceived, the next monofilament is tested. The tactile sensation threshold was determined by the thinnest monofilament they could feel (Unver and Akbas, 2018).

### 2.6 Strength test

The strengths of knee flexion and extension on the affected leg were measured using the IsoMed 2000 strength testing system (D & R Ferstl GmbH, Hemau, Germany; Figure 1D), which showed good test-retest reliability (ICC = 0.77–0.98) (Gonosova et al., 2018). Participant were seated on the dynamometer chair with their knee and hip placed at about 90° and 85°, respectively. Their torso, pelvis, and thigh were secured to the training chair using a lap belt. The chair was adjusted to align each participant’s lateral femoral condyle with the axis of rotation of the dynamometer arm and the distal torque pad was affixed to the shank at two finger-widths above the lateral malleolus. The position of the safety valve was adjusted to limit the range of joint movement to prevent accidents and ensure the participant’s safety. All participants were instructed to flex and extend the tested leg with the maximal exertion at an angular speed of 60°/s. The reliability at this angular velocity was high (0.87 < ICC < 0.96) (Roth et al., 2017). Three trials were recorded, and at least a 2 min break was taken between two trials. Max knee flexion and extension torques were normalized by body mass.

### 2.7 Data analysis

Descriptive analysis was used to summarize the means and standard deviations of RTS variables, strength, proprioception, and tactile sensation. The normality of all outcome variables was tested using Shapiro–Wilk tests. A partial correlation (Pearson correlation for normally distributed or Spearman correlation for non-normally distributed data) was used to verify Hypothesis 1 by determining the correlations of the RTS variables with each of the strength, proprioception, and tactile sensation variables while controlling for covariates (age, height, weight, and postoperative duration). Then, a separate exploratory factor analysis was carried out among each category of the variables of interest. Multivariable linear regression was used to verify Hypothesis 2 by exploring the degrees of correlation between each generated factor and RTS variables while controlling for the above-mentioned covariates. The thresholds for the correlation coefficient (r) were as follows: 0–0.1, trivial; 0.1–0.3, weak; 0.3–0.5, moderate; >0.5, strong (Cohen, 1988). All analyses were conducted in SAS 9.4, and the significance level was set at 0.05.

## 3 Results

Shapiro–Wilk tests showed that most of the tactile sensation and ACL-RSI data were non-normally distributed. Strength, proprioception, K-STARTS, and functional performance data were all normally distributed.

The descriptive characteristics are shown in Table 1. Mean, standard deviation, and minimum and maximum values are reported for the RTS variables, strength, proprioception, and tactile sensation.

**TABLE 1:** Descriptive characteristics of outcome variables.

		Mean	SD	Max	Min
RTS variables	K-STARTS	10.45	3.24	16.00	2.00
	ACL-RSI	1.55	0.86	3.00	0.00
	Functional performance	8.90	3.12	14.00	2.00
Strength (N·m/kg)	Knee flexion	1.15	0.49	2.33	0.48
	Knee extension	0.87	0.51	1.82	0.16
Proprioception (°)	Knee flexion	0.92	0.32	1.55	0.27
	Knee extension	0.90	0.35	1.66	0.22
Cutaneous sensitivity (gauge)	Great toe	3.60	0.40	4.31	2.83
	1st metatarsal	3.35	0.52	4.31	2.83
	5th metatarsal	3.64	0.53	4.31	2.83
	Arch	3.65	0.44	4.31	2.83
	Heel	3.75	0.41	4.31	2.83

RTS: return to sports; K-STARTS: knee santy athletic return to sports; ACL-RSI: anterior cruciate ligament return to sports after injury scale.

Partial correlations are shown in Table 2. Knee flexion strength was moderately correlated with K-STARTS total and functional scores, and knee extension strength was moderately correlated with K-STARTS total, ACL-RSI, and functional scores. Knee flexion proprioception was moderately correlated with K-STARTS total and functional scores, and knee extension proprioception was moderately correlated with K-STARTS total and functional scores. Tactile sensation at the fifth metatarsal was moderately correlated with K-STARTS total and functional scores.

**TABLE 2:** Partial correlation outcomes of K-STARTS, ACL-RSI, and functional performance with strength, proprioception, and tactile sensation variables.

		K-STARTS		ACL-RSI		Functional performance	
	Variables	*r*	p	*r*	p	*r*	p
Strength (N·m/kg)	Knee flexion	0.340	0.032	−0.074	0.648	0.374	0.017
	Knee extension	0.415	0.008	0.358	0.023	0.327	0.040
Proprioception (°)	Knee flexion	−0.316	0.047	0.092	0.571	−0.355	0.025
	Knee extension	−0.321	0.044	0.028	0.864	−0.340	0.032
Tactile sensation (gauge)	Great toe	0.220	0.172	0.102	0.532	0.199	0.218
	1st metatarsal	−0.198	0.221	0.243	0.13	−0.276	0.085
	5th metatarsal	−0.395	0.012	0.057	0.725	−0.426	0.006
	Arch	0.059	0.717	0.186	0.252	0.008	0.963
	Heel	0.011	0.948	0.176	0.276	−0.040	0.806

RTS: return to sports, K-STARTS: knee santy athletic return to sports, ACL-RSI: anterior cruciate ligament return to sports after injury scale. The correlations of tactile sensation to ACL-RSI, were analyzed by Spearman correlation. The correlations of the others variables were analyzed by Pearson correlation. The shaded cells represent significant correlation coefficients. The values were adjusted for age, weight, height, and postoperative duration. *r*: correlation coefficient.

The factor loadings for all the variables of strength, proprioception, and tactile sensation are shown in Table 3. Factor 1 (F1), factor 2 (F2), and factor 3 (F3) were the summaries of strength, proprioception, and tactile sensation, respectively, with a Kaiser Meyer Olkin value of 0.760 and sphericity of <0.001.

**TABLE 3:** Factor loadings for the variables among the categories of joint torque, proprioception, and tactile sensation.

		Factor 1	Factor 2	Factor 3
Strength (N·m/kg)	Knee flexion	0.518	--	--
	Knee extension	0.880	--	--
Proprioception (°)	Knee flexion	--	0.793	--
	Knee extension	--	0.759	--
Tactile sensation (gauge)	Great toe	--	--	0.636
	1st metatarsal	--	--	0.798
	5th metatarsal	--	--	0.733
	Arch	--	--	0.719
	Heel	--	--	0.779

The equations for multivariable regression are:

$$K-STARTS\;total\;score=10.452+\left(1.564\times F1\right)-\left(0.869\times F2\right)$$ (1)

$$Functional\;score=8.905+\left(1.369\times F1\right)-\left(1.038\times F2\right)$$ (2)

In Eq. 1, adjusted *r* ^2^ = 0.517, p_F1_ = 0.001, p_F2_ = 0.048, *β*_F1_ = 0.483, and *β*_F2_ = 0.268. In Eq. 2, adjusted *r* ^2^ = 0.520, p_F1_ = 0.002, p_F2_ = 0.015, *β*_F1_ = 0.439, and *β*_F2_ = 0.332. No significant correlations were detected between ACL-RSI and the three factors.

The equations indicated that compared with proprioception, strength has more contribution to K-STARTS total (*β*_F1_ = 0.483>*β*_F2_ = 0.268) and functional scores (*β*_F1_ = 0.439>*β*_F2_ = 0.332).

## 4 Discussion

This study investigated the correlations of strength, proprioception, and tactile sensation with RTS readiness. The outcomes partly supported Hypothesis 1 that knee strength is correlated with K-STARTS total, ACL-RSI, and functional scores. While proprioception and tactile sensation were only correlated with K-STARTS and functional scores, and not with ACL-RSI. The outcomes supported Hypothesis 2 and pointed out that strength contributed more to K-STARTS total and functional scores than proprioception and that tactile sensation had the least contribution.

The results showed that strength is correlated with K-STARTS total, functional, and ACL-RSI scores. The correlation between strength and functional performance was consistent with previous studies (Myer et al., 2011; Xergia et al., 2015) but inconsistent with another study (Barber et al., 1990). The inconsistencies may be attributed to the different angular velocities in the strength tests. The angular velocity during strength tests was 300°/s in Barber et al.’s study, while 60°/s in the present study. It has been confirmed that isokinetic torque at 60°/s provides valuable information on strength recovery after ACLR (Eitzen et al., 2009). Previous studies supported our outcomes by pointing out that insufficient strength in the quadriceps femoris and hamstrings results in a high risk of re-injury (Callaghan and Oldham, 2004), our study showed a correlation between strength and K-STARTS, which may imply that strength plays an important role in RTS overall readiness. Furthermore, our outcomes also pointed out that knee extension strength was moderately correlated with ACL-RSI. Consistent with our observations, several studies showed a positive correlation between the strength of the affected quadriceps and the psychological readiness, greater quadriceps strength may lead to better confidence in performing functional activities (Lepley et al., 2018). Previous literature has shown that psychological readiness is an important determinant of RTS decisions (Ardern et al., 2014a), and vital element to RTS (Gokeler et al., 2017). Growing evidence supports the impact of psychological dysfunction on RTS among patients with ACLR (Burland et al., 2018). Our outcomes further imply that greater knee strength may enhance confidence in completing specific motor tasks and increase patients’ RTS confidence.

Our outcomes indicated that proprioception is correlated with K-STARTS total and functional scores. Our study demonstrates a correlation between proprioception and K-STARTS total score, which may imply that proprioception may influence RTS overall readiness. The correlation between proprioception and the functional score is consistent with some studies (Katayama et al., 2004; Chaput et al., 2022) but inconsistent with another study (Muaidi et al., 2009). The different choices of functional tests may explain the conflict. In the inconsistent study (Muaidi et al., 2009), only a single-legged hop-for-distance test was used. The ACL is a vital sensory organ that contains a variety of sensory nerve endings, provides proprioceptive information, generates protective reflexes, and plays an essential role in stabilizing the knee joint (Bonfim et al., 2003). When the knee flexed or extended, the tension of the surrounding muscles, tendons, and ligaments changes, which excites the mechanoreceptors and transmits information about joint motion and deformation to the CNS; then, the CNS modulates the corresponding muscles around the knee joint through reflex neuromuscular feedback, causing specific muscles to contract and thus maintaining joint stability (Roijezon et al., 2015). Previous studies pointed out that although the mechanical stability of the ACL can be restored in a short period of time, proprioceptive reconstruction takes longer (Lee et al., 2015). Our outcomes support that proprioception is another key to RTS and that proprioception-related rehabilitation should be included in ACLR programs.

Our outcomes also pointed out that tactile sensation at the 5th metatarsal head on the affected side is related to K-STARTS total and functional scores. This finding may be due to the compensation mechanism of tactile sensation with proprioception. Peripheral sensory signals are transmitted along different sensory neurons, such as large type Ia and II sensory neurons responsible for proprioception and small-diameter type III sensory neurons for tactile sensation (Li et al., 2019). Type III sensory neurons are slower and weaker than type Ia and II sensory neurons. Hence, individuals usually rely on proprioception rather than tactile sensation in neuromuscular control during dynamic movement (Song et al., 2021). Proprioception usually decreases with ACLR, and participants may use tactile sensation to compensate for their declined proprioception. This viewpoint is supported by previous studies that observed the compensation between the two senses (Li et al., 2019). Moreover, Impaired balance in the lateral direction is associated with a higher fall risk than impaired anterior-posterior balance (Islam et al., 2004). During locomotion, if the disturbance occurs in the anterior, posterior, or medial direction, the individual can counteract the disturbance by dropping the swinging leg in the appropriate position. However, if the disturbance occurs in the lateral direction, the individual may need the weak lateral foot muscles to maintain balance since it is difficult to drop the swing leg on the lateral of the supporting leg (Mao et al., 2006). It is inferred that the tactile sensation at the lateral part of foot sole, represented by the 5th metatarsal head, is more important than other parts to maintain balance and to facilitate functional performance. To the best of our knowledge, no studies have investigated the correlations of tactile sensation to RTS readiness, and the present study suggests that approaches to increase tactile sensation should be applied to ACLR rehabilitation at least when their proprioception is not well recovered.

Multivariate linear regression revealed that strength contributed more to RTS readiness than proprioception, and tactile sensation contributed the least. ACLR reduces quadriceps strength and impairs knee proprioception (Lepley et al., 2018). It has been pointed out that the recovery of lower extremity strength has a remarkable impact on the RTS of patients in ACLR(Kaya et al., 2019), and proprioceptive training decreases the incidence of ACL injury (Dargo et al., 2017). Our outcomes supported these viewpoints and filled the gap between previous studies by pointing out that strength has higher levels of correlation with RTS than proprioception, which infers that strength training has priority over proprioception rehabilitation in patients with ACLR.

This study has several limitations. First, most of the participants were male, which affects the applicability of the outcomes to female. The MICODT score may be influenced by gender. Second, this study used a passive proprioceptive assessment, and the results might have been different if an active assessment had been used. However, it has been pointed out that participants’ performance on active proprioception (usually measured using joint position sense) was more erratic overall compared with passive proprioception (usually measured using threshold to detect passive motion) (Reider et al., 2003), and passive proprioception assessment has better consistency in detecting proprioceptive defects than active assessment in ACL-deficient knees (Friden et al., 1997). Third, the participants had different types of sports, with ball games being the majority, which may have influenced our results.

## 5 Conclusion

Rehabilitation to promote muscle strength, proprioception, and tactile sensation should be performed among patients with ACLR, muscle strength has the highest priority, followed by proprioception, with tactile sensation making the least contribution to RTS among patients with ACLR.

## Data Availability

The datasets presented in this study can be found in online repositories. The names of the repository/repositories and accession number(s) can be found below: 10.57760/sciencedb.01729.
